# Increasing plant diversity enhances soil organic carbon storage in typical wetlands of northern China

**DOI:** 10.3389/fpls.2024.1467621

**Published:** 2024-12-02

**Authors:** Hua-Bing Liu, Li-Ping Yang, Jun-Qin Gao, Qian-Wei Li, Xing-Li Li, Jiu-Ge Feng, Fei-Hai Yu

**Affiliations:** ^1^ School of Ecology and Nature Conservation, Beijing Forestry University, Beijing, China; ^2^ The Key Laboratory of Ecological Protection in the Yellow River Basin of National Forestry and Grassland Administration, Beijing, China; ^3^ Guangdong Wetland Conservation Association, Guangzhou, China; ^4^ Institute of Wetland Ecology & Clone Ecology/Zhejiang Provincial Key Laboratory of Evolutionary Ecology and Conservation, Taizhou University, Taizhou, Zhejiang, China

**Keywords:** microbial biomass carbon, plant species diversity, soil organic carbon, semi-arid region, wetland ecosystems

## Abstract

Soil organic carbon plays an important role in climate change mitigation, and can be strongly affected by plant diversity. Although a positive effect of plant diversity on soil organic carbon storage has been confirmed in grasslands and forests, it remains unclear whether this effect exists in wetlands. In this study, we investigated plant diversity, soil properties and soil organic carbon across five typical wetlands of northern China, to test the effect of plant diversity on soil organic carbon and clarified the regulators. Increasing plant diversity significantly increased belowground biomass of wetland plant communities, and both soil organic carbon content and storage were significantly positively related to wetland plant diversity. The positive effect of plant diversity was influenced by belowground biomass of wetland plant communities, soil microbial biomass carbon, and soil properties, especially soil water content and bulk density. The structural equation model showed that soil organic carbon storage was dominantly affected by microbial biomass carbon, plant diversity and biomass, with standardized total effects of 0.66 and 0.47, respectively, and there was a significant positive relationship between soil organic carbon and microbial biomass carbon. These results suggest that increasing plant diversity can potentially promote the ability of wetlands to store organic carbon in soils. The findings highlight the importance of plant diversity on soil organic carbon in wetland ecosystems, and have implications for managing wetlands to increase carbon sinks and to mitigate global climate change.

## 1 Introduction

Wetland ecosystems stored more than 30% of terrestrial soil organic carbon (SOC) despite occupying less than 6% of land area (Poulter et al., 2021). As one of the highest carbon sinks and carbon-dense ecosystems, wetland ecosystems can significantly affect global carbon cycling (Zhang et al., 2023). Plant diversity plays an important role in community structuring and ecosystem functioning such as carbon sequestration (Hector et al., 1999; Xue et al., 2022; Huang et al., 2023; Liu et al., 2024). Increasing plant diversity has been shown to enhance soil organic carbon storage (SOCS) in forests (Chen et al., 2023), grasslands (Steinbeiss et al., 2008; Cong et al., 2014; Chen et al., 2018), and agricultural ecosystems (Chen et al., 2020). However, the relationship between plant diversity and SOCS in natural wetland ecosystems remains unknown. Understanding such a relationship can potentially help manage wetland ecosystems to promote carbon sequestration and thus to mitigate global climate change.

Plant diversity can influence SOCS through plant biomass and plant-derived carbon inputs (Gould et al., 2016; Li et al., 2022; Xi et al., 2023), because high plant diversity may promote the complementarity among species and the selection of some highly productive plant species (Loreau and Hector, 2001; Allan et al., 2011; Adomako et al., 2019; Wang X. et al., 2022). Previous studies have confirmed that increasing plant diversity promotes plant biomass (Chen et al., 2018; Bai et al., 2021; Wang et al., 2023), although there are still inconsistent conclusions (Gherardi and Sala, 2015; Zou et al., 2024). Plant diversity can affect SOCS by influencing soil labile organic carbon and soil properties in forest ecosystems (Ratcliffe et al., 2017; Adair et al., 2018). Additionally, increasing plant diversity has been found to promote dissolved organic carbon (DOC) and microbial biomass carbon (MBC) (Thakur et al., 2015; Mellado-Vazquez et al., 2016; Lange et al., 2021), thereby increasing SOCS in grassland ecosystems (Prommer et al., 2020). For wetland ecosystems, plant diversity can also impact SOC accumulation through changing plant detritus inputs (Gould et al., 2016; Li et al., 2022; Xi et al., 2023). A previous study demonstrated that root biomass was significantly correlated with plant richness in freshwater wetlands (Schultz et al., 2011). Thus, it is likely that there is also a positive effect between plant diversity and SOCS in wetland ecosystems. The effect of plant diversity on SOCS can vary with environmental conditions such as climate (Spohn et al., 2023). In semi-arid grasslands, for instance, mean annual temperature (MAT) and precipitation (MAP) significantly regulate the effect of plant diversity on SOCS (Tian et al., 2016). Furthermore, in wetland ecosystems, there were significant correlations between plant diversity and soil properties, which maybe complexly drive SOC sequestration (Ma et al., 2021; Zhao et al., 2021). Increasing plant diversity may enhance SOCS through increasing the availability of labile carbon from plants to microbes (Lange et al., 2015; Mellado-Vazquez et al., 2016; Chen et al., 2018; Prommer et al., 2020). Previous studies have demonstrated that increased plant litter inputs provide more substrate for soil microorganisms, enhancing SOCS through increased microbial residue accumulation (Eisenhauer et al., 2010; Liang et al., 2011; Lange et al., 2015). Additionally, high plant richness and biomass have been shown to increase microbial enzyme activity in wetlands, which maybe affect SOCS, similar to those observed in other terrestrial ecosystems (Kim et al., 2022). Moreover, SOCS is also significantly affected by soil properties, i.e., the soil water content and pore size distribution (Fukumasu et al., 2022). As these biotic and abiotic factors are closely linked with SOCS, increasing plant diversity would theoretically enhance SOCS in wetland ecosystems. However, few studies have tested the potential mechanisms underlying the relationship between plant diversity and SOCS in wetlands.

Here, we conducted a field experiment in five typical wetlands (Daihai, Chagannor, Dalinor, Horqin and Wulagai) in a semi-arid region of northern China. We tested the effects of increasing plant diversity on SOCS and biomass, and clarified the key determinant regulating SOCS. Given that SOCS is influenced not only by plant carbon input but also by soil properties and mineralization processes, we hypothesized that: (1) increasing plant diversity would increase plant community biomass, and thereby enhance SOC and its fractions (DOC and MBC) in wetlands; (2) SOCS is jointly regulated by plant diversity and biomass, soil properties such as soil water content and MBC.

## 2 Materials and methods

### 2.1 Site description

**Figure 1:**
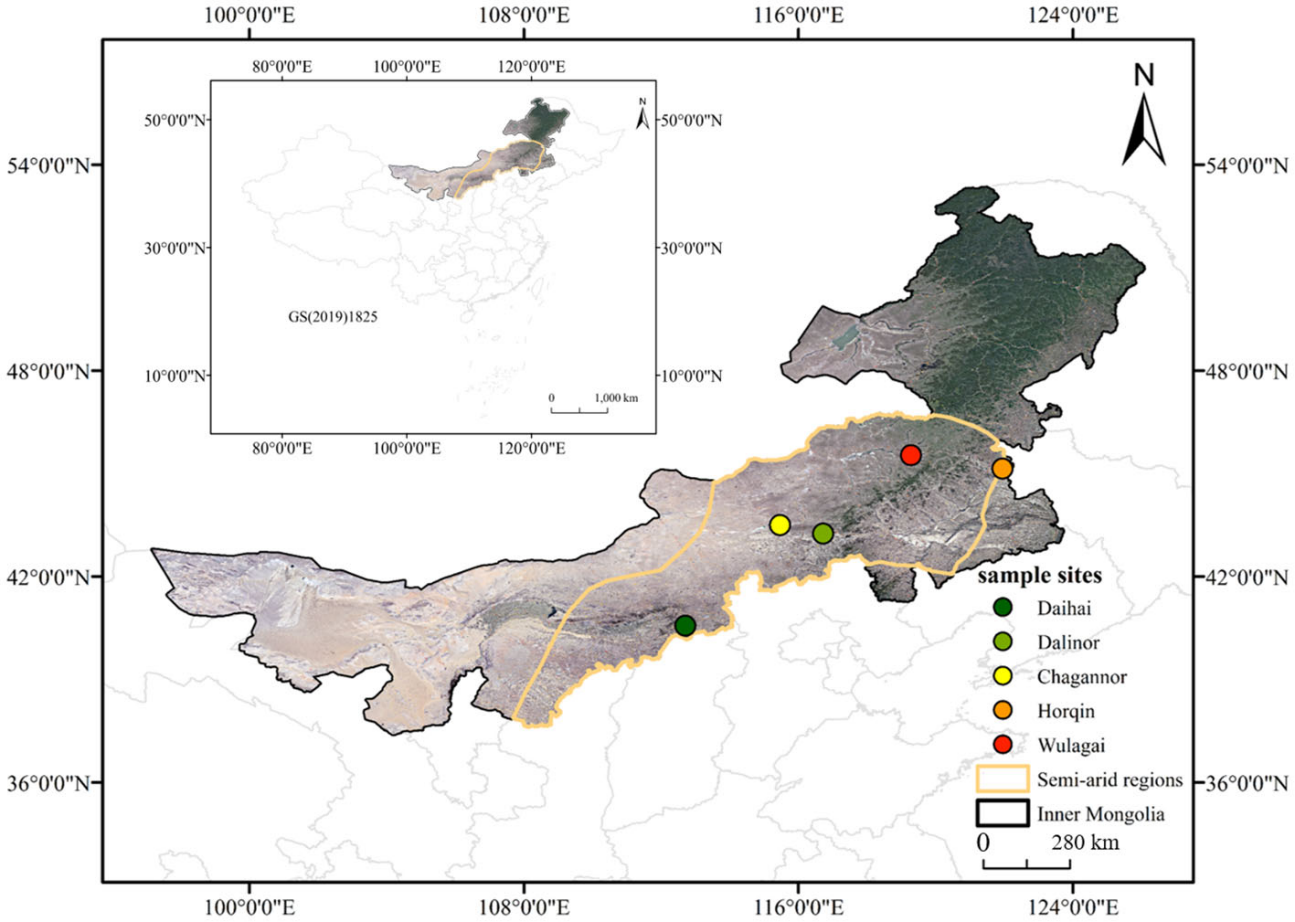
Study area and detailed location of the sampling sites.

We selected five typical wetlands (Daihai, Chagannor, Dalinor, Horqin and Wulagai) with minimal anthropogenic interference in Inner Mongolia, semi-arid region in northern China (40°13’-45°54’ N, 115°27’-121°57’ E; 179 m-1242 m a.s.l.; **Figure 1**). This region has a typical continental monsoon climate, with MAP of approximately 350-500 mm and MAT of around 3-7 °C (Yao et al., 2023). The growing season spans from May to September, with about 70% of the yearly rainfall occurring during June to August (Wu et al., 2010; Fang et al., 2018). The dominant plant species are *Equisetum hyemale*, *Leymus chinensis*, *Potentilla anserina* and *Phragmites australis* (**Table 1**).

**Table 1:** Mean annual precipitation (MAP), mean annual temperature (MAT) and plant communities at sampling sites.

Site	MAP (mm)	MAT (°C)	Longitude (°N)	Latitude (°W)	Plant species
Daihai	450.56	6.71	121.95	45.14	*Phragmites australis*, *Cyperus pygmaeus*, *Schoenoplectus tabernaemontani*, *Schoenoplectus triqueter*, *Suaeda salsa*, *Alisma plantago-aquatica*, *Knorringia sibirica*, *Aster tataricus*, *Inula japonica*, *Typha orientalis*
Chgannor	410.49	2.95	115.46	43.49	*Phragmites australis*, *Agropyron cristatum*, *Potentilla anserina*, *Artemisia*, *Ranunculus japonicus*, *Leymus chinensis*, *Scirpus triqueter*, *Saussurea amara*
Dalinor	432.11	3.76	116.72	43.25	*Suaeda salsa*, *Phragmites australis*, *Potentilla anserina*, *Parnassia palustris*, *Inula japonica*, *Cyperus rotundus*, *Melilotus officinalis*, *Calamagrostis epigeios*
Horqin	466.67	6.44	112.63	40.57	*Phragmites australis*, *Equisetum hyemale*, *Leymus chinensis*, *Artemisia*, *Lespedeza bicolor*, *Echinochloa crusgalli*, *Poaceae*, *Setaria viridis*, *Gramineae*, *Allium senescens*, *Parthenocissus Planch*
Wulagai	532.45	1.67	119.49	45.90	*Phragmites australis*, *Polygonum hydropiper*, *Vicia sepium*, *Cicuta virosa*,*Lactuca indica*, *Equisetum hyemale*, *Potentilla anserina*, *stertataricus*, *Sanguisorba officinalis*, *Swertia bimaculate*, *Poaceae Gramineae*

### 2.2 Field surveys and experimental analyses

In each wetland, we set up 12 plots of 2 m × 2 m with different plant species richness, totaling 60 plots for all five wetlands. In each plot, we recorded the name of each plant species, measured its height, and counted its individuals (**Table 1**). We then harvested aboveground and belowground parts in a randomly selected 20 cm × 20 cm quadrat in each plot.

In each plot, we collected soil samples from 0-10 cm, 10-20 cm and 20-30 cm soil depth using the five-point sampling method for physicochemical analysis. We also collected three soil cores (100 cm^3^) at each depth in each plot to measure soil water content and bulk density. Plant and soil samples were placed in self-sealing bags and transported to the laboratory. Plant samples were oven-dried at 65°C for 48 h, and weighed to obtain aboveground biomass and belowground biomass. One half of each soil sample was stored at -18 °C for subsequent measurements of DOC and MBC. The other half of the soil sample was air-dried at room temperature and then sieved through a 2-mm sieve. We measured pH and electrical conductivity (EC) using a pH meter (PHB-3, China) and a conductivity meter (FE30, USA) with a soil:water ratio of 1:5 (w:w), respectively (Zhao et al., 2020). DOC, MBC and SOC were measured by TOC-analyzer (N/C 3100, Germany). Specifically, DOC was extracted with a mass ratio of 1:5 (soil/purified water) and subsequently analyzed by TOC-analyzer (N/C 3100, Germany). MBC was determined by the chloroform fumigation method with TOC-analyzer (N/C 3100, Germany) (Vance et al., 1987). SOC was measured using a high-temperature combustion method with the solid module of TOC-analyzer (N/C 3100, Germany) after inorganic carbon was removed (Bisutti et al., 2004; Liang et al., 2021).

### 2.3 Calculations

Diversity indices (species richness, Shannon-Wiener index, and Simpson’s dominance index) were used to characterize plant species diversity (Fang et al., 2009).

Shannon-Wiener index (H) measures the degree of diversity in plant communities (Shannon and Weaver, 1949) and was calculated as:


(1) $$\text{H}=-\sum_{i=1}^{s}Pi\ln\left(Pi\right)$$

where *S* represents the total number of plant species in a given plot, *P_i_* represents the relative abundance of plant species *i* within that plot.

Simpson’s dominance index (D) reflects evenness in distribution among species (Simpson, 1949) and was calculated as:


(2) $$\text{D}=1-\sum_{i=1}^{s}{\left(ni/N\right)}^{2}$$

The variable *ni* represents the number of individuals belonging to plant species *i*, while *N* is the total number of individuals across all plant species within a given plot.

Soil organic carbon storage (SOCS, kg·m^-2^) in a given soil layer (Satdichanh et al., 2023) was calculated as:


(3) $$\text{SOCS}={\text{SOC}}_{i}\;\times {\text{BD}}_{i}\;\times \text{H}\times \left(1-\text{CF}/100\right)\times 0.01$$

where SOC*_i_* is SOC contents of the soil layer *i* (mg·g^-1^), BD*_i_* is the soil bulk density of soil layer *i* (g·cm^-3^), H is the layer thickness (cm), CF is the percentage of coarse fragments > 2 mm, and 0.01 is a unit conversion coefficient.

### 2.4 Statistical analyses

Regression analyses was used to explore the relationships between plant diversity (species richness, H and D), plant biomass (total biomass, aboveground biomass, and belowground biomass) and soil carbon (DOC, MBC, SOC and SOCS). Random forest model (RF) was used to explore the relative effects of each variable on SOC. We used the “rfPermute” package to determine the significance of variables (Jiao et al., 2018). The importance of factors was estimated by calculating the percentage increases in the MSE (mean squared error) of variables. For instance, higher values of MSE% indicate more significant variables (Breiman, 2001). Based on the expected relationship between SOC and potential drivers as well as the results of the RF analysis, we developed a structural equation model (SEM) to analyze the direct and indirect SOC determinants by using the “lavaan” package. Path diagrams are commonly used in SEM analysis to illustrate directional relationships between different variables. We used the maximum likelihood method to estimate path coefficients, and fit indices were utilized to evaluate model fit (χ^2^/df<  3, GFI  >  0.9 and RMSEA< 0.08). All statistical analyses were conducted using R 4.2.1 (https://www.r-project.org/).

## 3 Results

### 3.1 Effects of plant diversity on soil organic carbon

**Figure 2:**
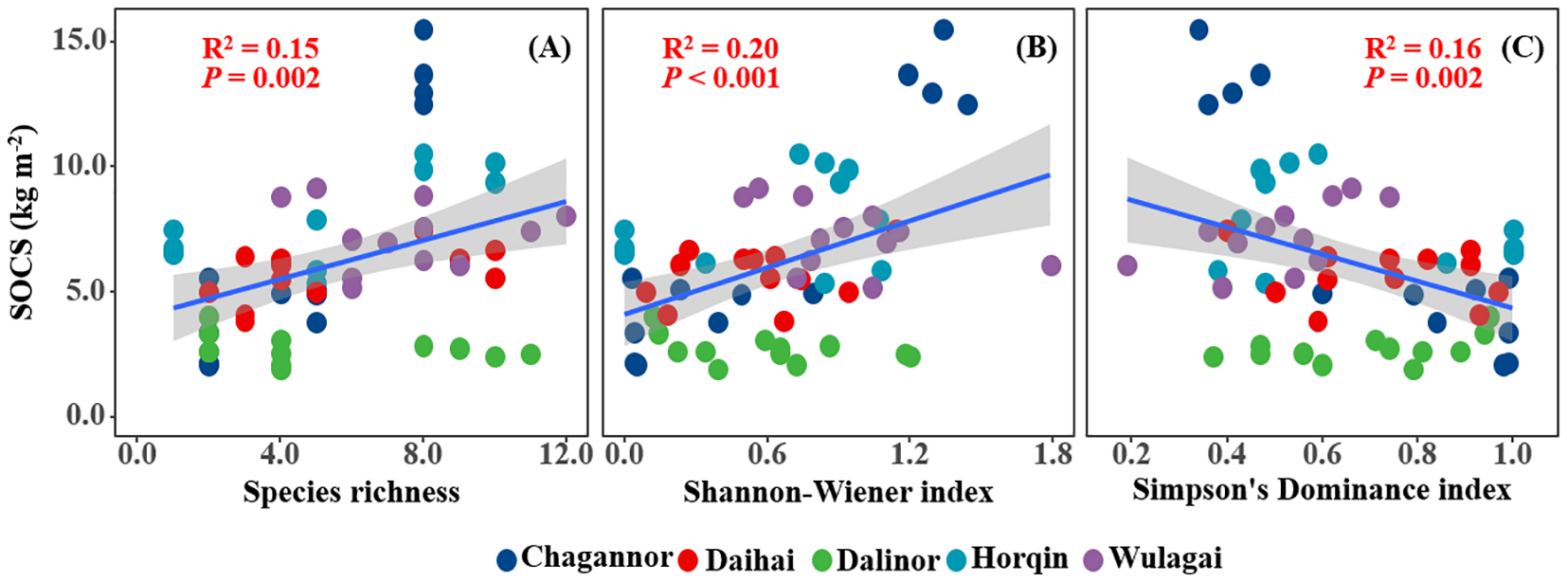
Relationships between plant diversity (Richness index, Shannon-Wiener index, Margalef index and Simpson's dominance index) and soil organic carbon storage (SOCS). R^2^, and *P*-values of linear regressions are presented, and the shaded areas represent the 95% confidence intervals (*P* < 0.05). SOCS, soil organic carbon storage.

**Figure 3:**
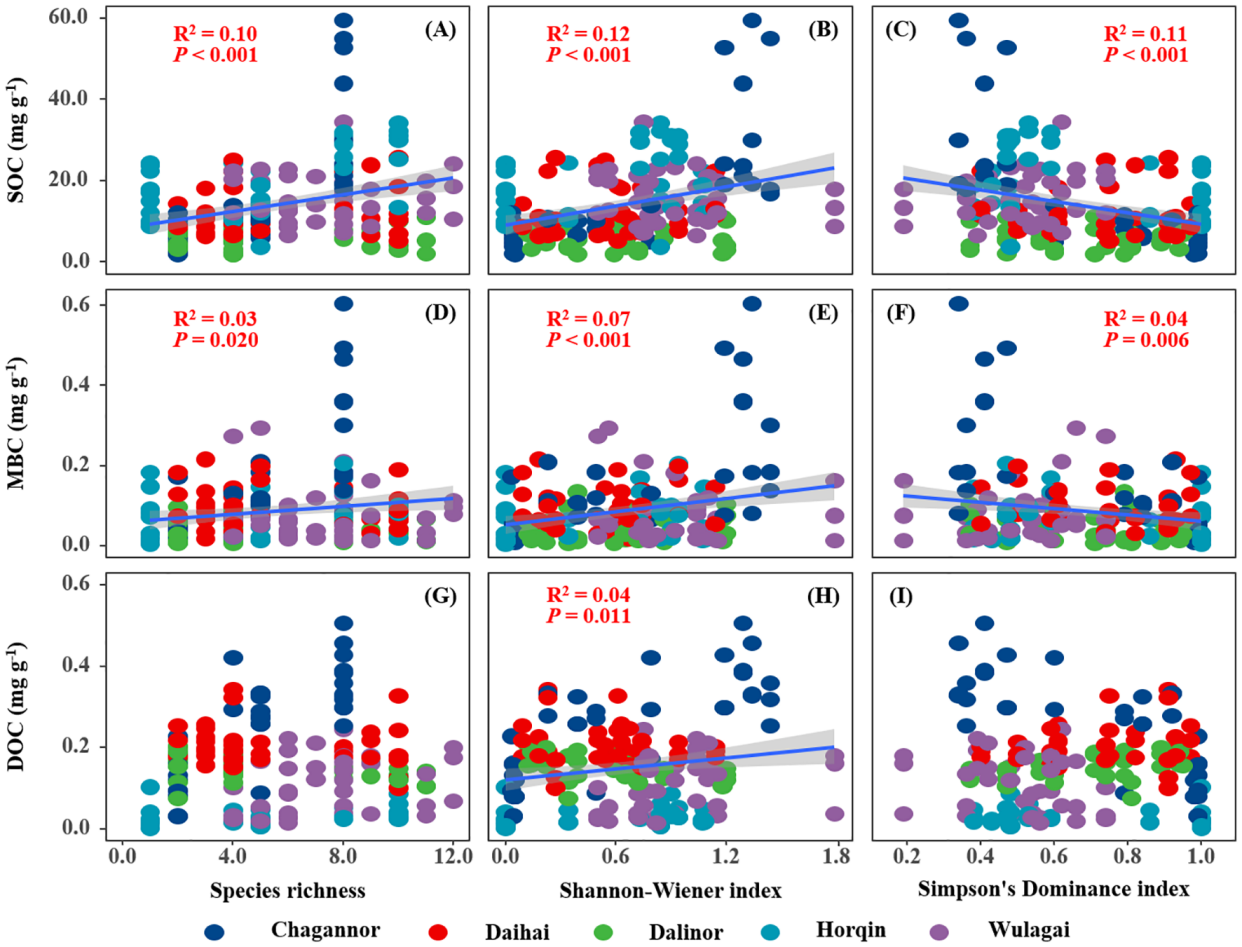
Relationships between plant diversity (Richness index, Shannon-Wiener index, Margalef index and Simpson's dominance index) and soil carbon (SOC, MBC and DOC). R^2^, and *P*-values of linear regressions are presented, and the shaded areas represent the 95% confidence intervals (*P* < 0.05). SOC, soil organic carbon; MBC, microbial biomass carbon; DOC, dissolved organic carbon.

SOCS of the wetland had a significant positive relationship with species richness and Shannon-Wiener index, but a negative relationship with Simpson’s dominance index (**Figures 2A-C**). Similarly, there was a significantly positive correlation between plant diversity (species richness and Shannon-Wiener index) and SOC (**Figures 3A, B**), MBC (**Figures 3D, E**). Simpson’s dominance index is significantly negatively related to SOC and MBC (**Figures 3C, F**). DOC had no significant relationship with species richness and Simpson’s dominance index, but a positive relationship with Shannon-Wiener index (**Figures 3G-I**).

### 3.2 Effects of plant diversity on community biomass

**Figure 4:**
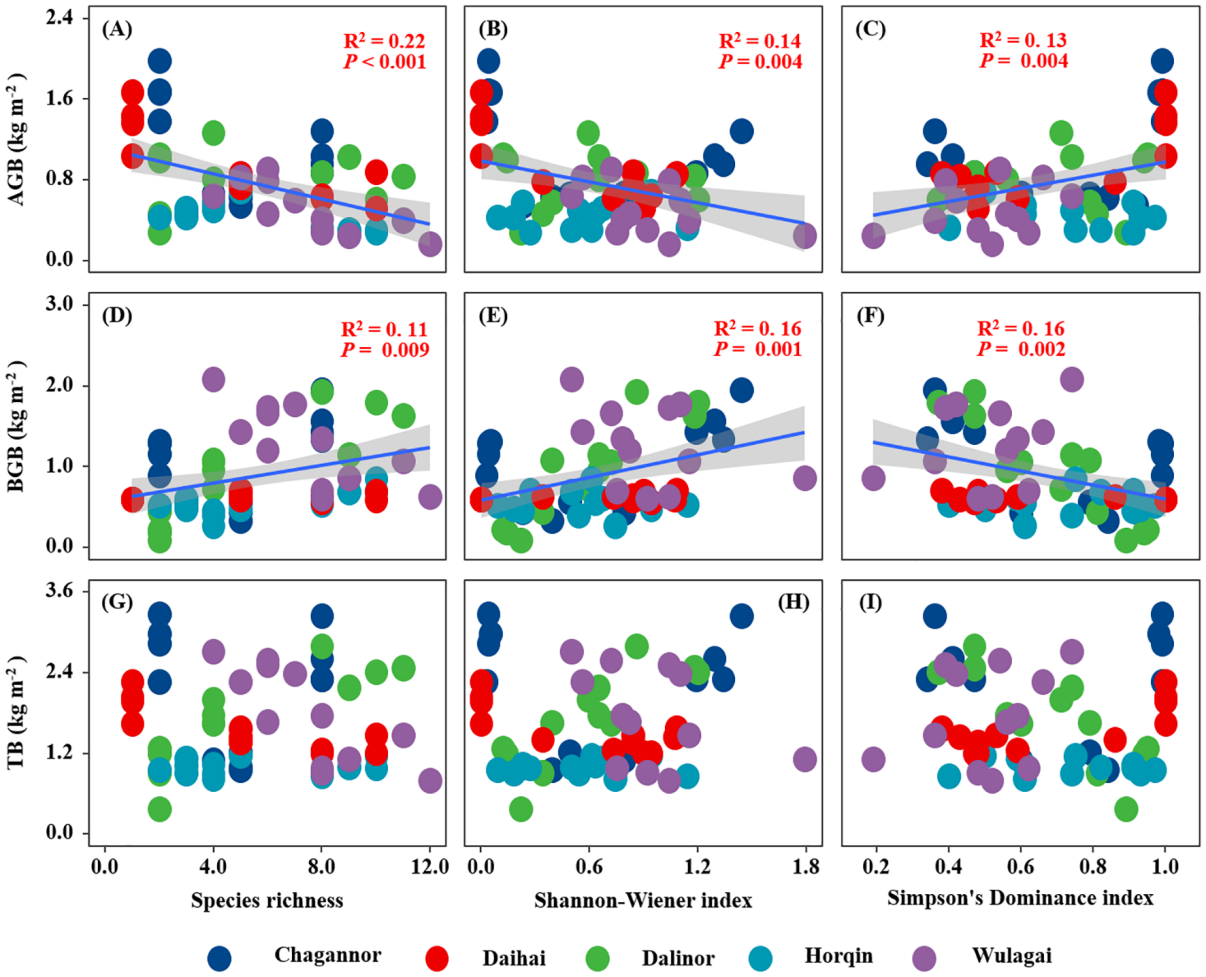
Relationships between plant diversity (Richness index, Shannon-Wiener index, Margalef index and Simpson's dominance index) and plant community biomass (AGB, BGB and TB). R^2^, and *P*-values of linear regressions are presented, and the shaded areas represent the 95% confidence intervals (*P* < 0.05). AGB, aboveground biomass; BGB, belowground biomass; TB, total biomass.

Belowground biomass of the wetland plant communities had a significant positive relationship with species richness and Shannon-Wiener index, but a negative relationship with Simpson’s dominance index (**Figures 4D-F**). Conversely, aboveground biomass of the wetland plant communities was significantly negatively related to species richness and Shannon-Wiener index and positively related to Simpson’s dominance index (**Figures 4A-C**). There was no significant relationship between plant diversity and total biomass of the wetland communities (**Figures 4G-I**).

### 3.3 Dominant determinants of SOC

**Figure 5:**
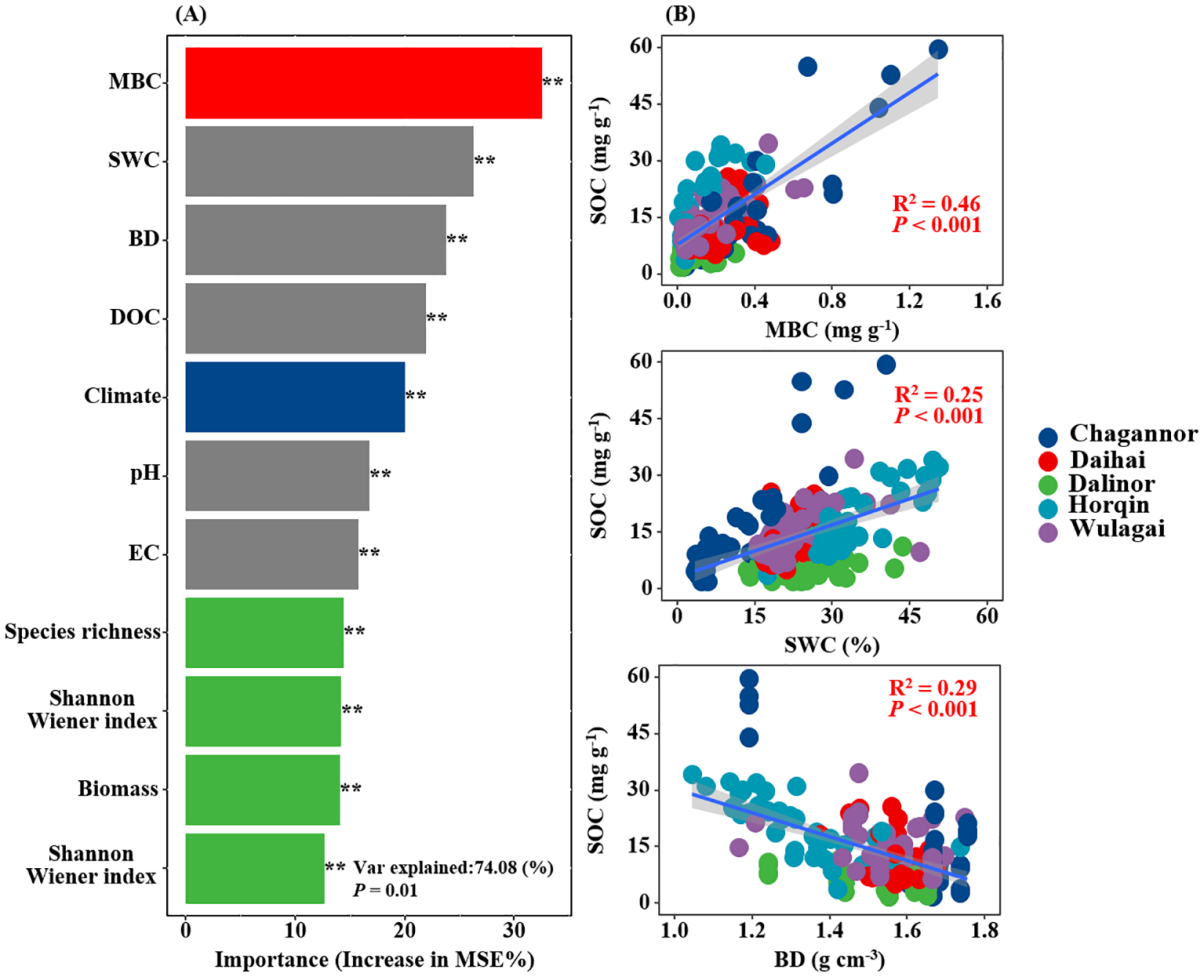
Relative importance of Climate, plant (species richness, Shannon-Wiener index, Simpson’s Dominance, and Biomass), soil (BD, SWC, EC, pH and DOC), as well as microorganisms (MBC) on SOC was measured by the percentage increase of the mean squared error (MSE%) using random forest (RF) models **(A)**. Relationships between SOC and MBC, BD and SWC **(B)**. MBC, microbial biomass carbon; BD, soil bulk density; SWC, soil water content; DOC, dissolved organic carbon; EC, electrical conductivity; SOC, soil organic carbon. R^2^, and *P*-values of linear regressions are presented, and the shaded areas represent the 95% confidence intervals (*P*< 0.05).

The results of RF analysis showed that MBC was the most important determinant influencing SOC, followed by soil bulk density and soil water content (**Figure 5A**). DOC, plant diversity and climate had similar contributions in regulating SOC across the study region. SOC was significantly and positively related to MBC (R^2^ = 0.46, *P<* 0.001), soil water content (R^2^ = 0.25, *P<* 0.001), significantly and negatively related to bulk density (R^2^ = 0.29, *P<* 0.001, **Figure 5B**).

**Figure 6:**
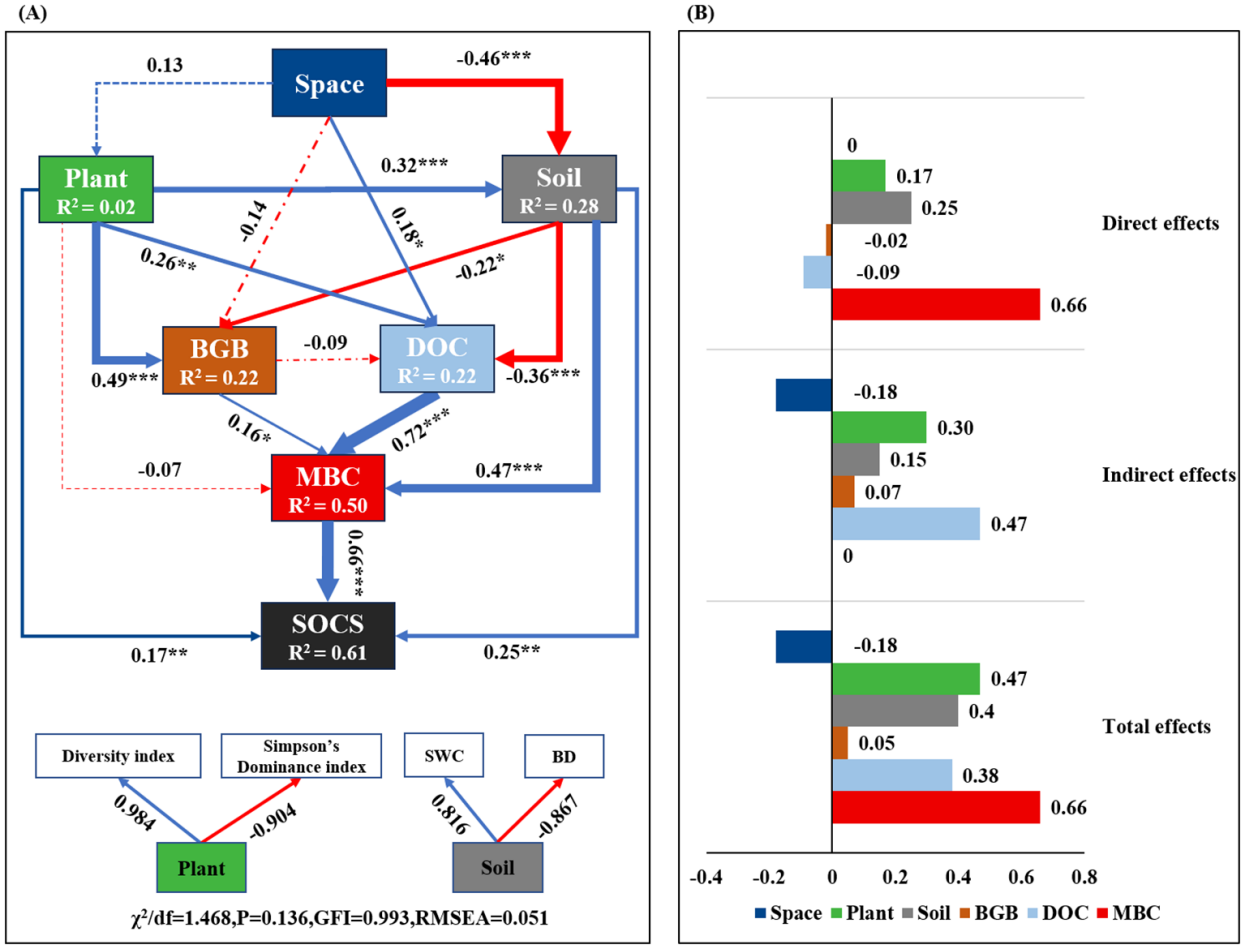
Structural equation modeling (SEM) for the effects of the variables on SOCS **(A)**, direct, indirect, and total standardized effects of each variable on SOCS from SEM **(B)**. The Plant and Soil were indicated by the first axis of principal component analysis (PCA). Space includes Sites and Climate. Plant includes Diversity index (species richness and Shannon-Wiener index) and Simpson’s Dominance index. Soil in this model includes SWC and BD. Numbers adjacent to arrows are standardized path coefficients (* *P*< 0.05, ** *P*< 0.01, *** *P*< 0.001), with dotted lines indicates non-significant pathways and solid lines indicates significant effects. The blue arrow indicates positive pathway, and the red arrow indicates negative pathway. The width of arrows is proportional to the strength of path coefficients, and indicative of effect size of the relationship. R^2^ denotes the proportion of variance explained. The goodness-of-fit (GFI) and root mean square error of approximation (RMSEA) are shown beside the model. BGB, belowground biomass; DOC, dissolved organic carbon; MBC, microbial biomass carbon; SOCS, soil organic carbon storage; SWC, soil water content; BD, soil bulk density.

The SEM analysis results showed that SOCS was dominantly and directly affected by MBC, soil, and plant, with standardized direct effects of 0.66, 0.25, and 0.17, respectively (**Figure 6**). Furthermore, plant and DOC indirectly affected SOCS, with standardized indirect effects of 0.30 and 0.47 (**Figure 6**). Moreover, soil water content and bulk density significantly and indirectly affected SOCS through a positive path of MBC (path coefficient = 0.47). Thus, increasing soil MBC resulted in enhanced SOCS with belowground biomass and soil properties changed (**Figure 6**).

## 4 Discussion

Plant diversity enhanced SOCS in typical wetlands of northern China, consistent with previous studies in forest (Chen et al., 2023), grassland (Steinbeiss et al., 2008; Cong et al., 2014), and agricultural ecosystems (Chen et al., 2018; Spohn et al., 2023). Previous studies indicated that plant diversity could indirectly affect SOCS by regulating soil labile carbon process (Chen et al., 2018; Lange et al., 2021). In our study, plant diversity significantly increased belowground biomass. This was consistent with previous studies which showed that diversity effect enhanced belowground productivity (Chen et al., 2018; Mylliemngap and Barik, 2019; Li et al., 2022; Zhou et al., 2022). Generally, different plant species exhibit diverse growth strategies and root structures, which promote the accumulation of belowground biomass through species complementarity (Loreau and Hector, 2001; Allan et al., 2011). However, we found that plant diversity significantly decreased aboveground biomass and had no significant effect on total biomass. Previous studies showed that plant diversity could promote aboveground biomass and total biomass in forests and grasslands (Mylliemngap and Barik, 2019; Token et al., 2022; Wang et al., 2023), which was not consistent with our results in wetlands. In natural wetlands, monodominant community with less plant species, such as *Phragmites australis*, tend to have high aboveground biomass, supported by the relationship between plant biomass and Simpson’s dominance index. Due to the opposite effects of plant diversity on aboveground and belowground biomass, there is no significant effect of plant diversity on total biomass in this study.

Our results showed that plant diversity increased soil carbon storage by increasing belowground biomass, DOC and MBC. Previous studies showed that plant biomass and MBC were significantly correlated with plant residues carbon and microbial necromass carbon, which were key sources crucial of SOC (Xu et al., 2023; Zheng et al., 2024). High plant diversity may increase the input of plant residues carbon by affecting the quantality and quality of plant litter, and promote the cumulation of microbial necromass carbon by regulating the structure and function of soil microbial community and MBC (Lange et al., 2015; Shen et al., 2022; Wang C. et al., 2022). In our study, both RF and SEM analyses demonstrated that MBC was the most significant determinant, and there was a significantly positive relationship between SOC and MBC (**Figure 5B**). This may be due to the fact that increased plant diversity with high belowground biomass and DOC transportation enhanced microbial activity (Dietrich et al., 2017), thereby promoting microbial-derived carbon contribution to SOCS through microbial turnover processes (Thakur et al., 2015; Prommer et al., 2020; Jia et al., 2021), consistent with a previous research (Lange et al., 2021). The positive effect of plant diversity on SOCS was also regulated by soil properties (Zhang et al., 2011; Yang et al., 2020). In our study, the relationship between plant diversity and SOCS might be related to the alternation of soil water content and bulk density. There was a significantly positive relationship between soil water content and SOC, and a negative relationship between bulk density and SOC, which was consistent with previous researches (Leimer et al., 2014; Fischer et al., 2015, 2019). Appropriate soil water content is beneficial for high plant diversity, enhancing plant residues carbon input, and also conducive to microbial decomposition; however, excessive soil water content can increase the anaerobic conditions in the soil, tend to form a single plant community, reduces the decomposition by aerobic microorganisms, thereby promoting carbon storage (Martinez Richart et al., 2019; Wang S. et al., 2022; Yan et al., 2020). Plant diversity may regulate soil water status by changing soil structure via plant roots, thus affecting SOC dynamics through microbial decomposition (Kammer et al., 2013; Wang et al., 2014; Ma et al., 2020; Lange et al., 2021). Therefore, how to promote SOC storage based on soil water regulation and plant diversity protection, and clarify the microbial mechanisms in wetland ecosystems still need further research. Given the nature of field surveys and sampling, it is possible that we may have overlooked the dynamic changes of plant diversity. In the future, long-term monitoring of plant diversity, coupled with systematic soil sampling, will be essential to reveal the complex mechanisms.

## 5 Conclusions

Plant diversity significantly enhanced SOC content and storage in typical wetlands of northern China by increasing belowground biomass and MBC, together with soil properties, especially soil moisture and bulk density. This study strengthens our knowledge about the positive effect of plant diversity on soil organic carbon storage in wetlands, and underscores the importance of considering plant diversity as a key component in the toolbox of wetland management and climate change mitigation strategies. Continued further research and long-term monitoring are necessary to understand the regulatory mechanism and management strategies of dynamic plant diversity and SOC storage under changing climatic conditions.

## Data Availability

The raw data supporting the conclusions of this article will be made available by the authors, without undue reservation.

## References

[B1] Adair E. C. Hooper D. U. Paquette A. Hungate B. A. (2018). Ecosystem context illuminates conflicting roles of plant diversity in carbon storage. Ecol. Lett. 21, 1604–1619. doi: 10.1111/ele.13145 30152093

[B2] Adomako M. O. Ning L. Tang M. Du D.-L. van Kleunen M. Yu F.-H. (2019). Diversity- and density-mediated allelopathic effects of resident plant communities on invasion by an exotic plant. Plant Soil. 440, 581–592. doi: 10.1007/s11104-019-04123-9

[B3] Allan E. Weisser W. Weigelt A. Roscher C. Fischer M. Hillebrand H. (2011). More diverse plant communities have higher functioning over time due to turnover in complementary dominant species. Proc. Natl. Acad. Sci. 108, 17034–17039. doi: 10.1073/pnas.1104015108 21949392 PMC3193239

[B4] Bai J. Meng Y. Gou R. Lyu J. Dai Z. Diao X.. (2021). Mangrove diversity enhances plant biomass production and carbon storage in Hainan island, China. Funct. Ecol. 35, 774–786. doi: 10.1111/1365-2435.13753

[B5] Bisutti I. Hilke I. Raessler M. (2004). Determination of total organic carbon – an overview of current methods. TrAC Trends Anal. Chem. 23, 716–726. doi: 10.1016/j.trac.2004.09.003

[B6] Breiman L. (2001). Random forests. Mach. Learn. 45, 5–32. doi: 10.1023/A:1010933404324

[B7] Chen X. Chen H. Y. H. Chen C. Ma Z. Searle E. B. Yu Z.. (2020). Effects of plant diversity on soil carbon in diverse ecosystems: a global meta-analysis. Biol. Rev. 95, 167–183. doi: 10.1111/brv.12554 31625247

[B8] Chen X. Taylor A. R. Reich P. B. Hisano M. Chen H. Y. H. Chang S. X. (2023). Tree diversity increases decadal forest soil carbon and nitrogen accrual. Nature 618, 94–101. doi: 10.1038/s41586-023-05941-9 37100916

[B9] Chen S. Wang W. Xu W. Wang Y. Wan H. Chen D.. (2018). Plant diversity enhances productivity and soil carbon storage. Proc. Natl. Acad. Sci. U. S. A. 115, 4027–4032. doi: 10.1073/pnas.1700298114 29666315 PMC5910804

[B10] Cong W.-F. Ruijven J. Mommer L. Deyn G. B. D. Berendse F. Hoffland E. (2014). Plant species richness promotes soil carbon and nitrogen stocks in grasslands without legumes. J. Ecol. 102, 1163–1170. doi: 10.1111/1365-2745.12280

[B11] Dietrich P. Buchmann T. Cesarz S. Eisenhauer N. Roscher C. (2017). Fertilization, soil and plant community characteristics determine soil microbial activity in managed temperate grasslands. Plant Soil 419, 189–199. doi: 10.1007/s11104-017-3328-4

[B12] Eisenhauer N. Beßler H. Engels C. Gleixner G. Habekost M. Milcu A.. (2010). Plant diversity effects on soil microorganisms support the singular hypothesis. Ecology 91, 485–496. doi: 10.1890/08-2338.1 20392013

[B13] Fang Q. Wang G. Liu T. Xue B.-L. Yinglan A. (2018). Controls of carbon flux in a semi-arid grassland ecosystem experiencing wetland loss: Vegetation patterns and environmental variables. Agric. For. Meteorol. 259, 196–210. doi: 10.1016/j.agrformet.2018.05.002

[B14] Fang J. Wang X. Shen Z. Tang Z. He J. Yu D.. (2009). Methods and protocols for plant community inventory. Biodivers. Sci. 17, 533–548. doi: 10.3724/SP.J.1003.2009.09253

[B15] Fischer C. Leimer S. Roscher C. Ravenek J. de Kroon H. Kreutziger Y.. (2019). Plant species richness and functional groups have different effects on soil water content in a decade-long grassland experiment. J. Ecol. 107, 127–141. doi: 10.1111/1365-2745.13046

[B16] Fischer C. Tischer J. Roscher C. Eisenhauer N. Ravenek J. Gleixner G.. (2015). Plant species diversity affects infiltration capacity in an experimental grassland through changes in soil properties. Plant Soil. 397, 1–16. doi: 10.1007/s11104-014-2373-5

[B17] Fukumasu J. Jarvis N. Koestel J. Kätterer T. Larsbo M. (2022). Relations between soil organic carbon content and the pore size distribution for an arable topsoil with large variations in soil properties. Eur. J. Soil Sci. 73, e13212. doi: 10.1111/ejss.13212

[B18] Gherardi L. A. Sala O. E. (2015). Enhanced precipitation variability decreases grass- and increases shrub-productivity. Proc. Natl. Acad. Sci. 112, 12735–12740. doi: 10.1073/pnas.1506433112 26417095 PMC4611653

[B19] Gould I. J. Quinton J. N. Weigelt A. Deyn G. B. D. Bardgett R. D. (2016). Plant diversity and root traits benefit physical properties key to soil function in grasslands. Ecol. Lett. 19, 1140–1149. doi: 10.1111/ele.12652 27459206 PMC4988498

[B20] Hector A. Schmid B. Beierkuhnlein C. Caldeira M. C. Diemer M. Dimitrakopoulos P. G.. (1999). Plant diversity and productivity experiments in European grasslands. Science 286, 1123–1127. doi: 10.1126/science.286.5442.1123 10550043

[B21] Huang L. Yao S.-M. Jin Y. Xue W. Lei N.-F. Chen J.-S.. (2023). Contrasting effects of species richness on soil pollutant removal in herbaceous plant communities: the importance of individual species. Restor. Ecol. 31, e13898. doi: 10.1111/rec.13898

[B22] Jia Y. Zhai G. Zhu S. Liu X. Schmid B. Wang Z.. (2021). Plant and microbial pathways driving plant diversity effects on soil carbon accumulation in subtropical forest. Soil Biol. Biochem. 161, 108375. doi: 10.1016/j.soilbio.2021.108375

[B23] Jiao S. Chen W. Wang J. Du N. Li Q. Wei G. (2018). Soil microbiomes with distinct assemblies through vertical soil profiles drive the cycling of multiple nutrients in reforested ecosystems. Microbiome 6, 146. doi: 10.1186/s40168-018-0526-0 30131068 PMC6104017

[B24] Kammer P. M. Schöb C. Eberhard G. Gallina R. Meyer R. Tschanz C. (2013). The relationship between soil water storage capacity and plant species diversity in high alpine vegetation. Plant Ecol. Divers. 6, 457–466. doi: 10.1080/17550874.2013.783142

[B25] Kim S. Kang H. Megonigal J. P. McCormick M. (2022). Microbial activity and diversity vary with plant diversity and biomass in wetland ecosystems. Estuaries Coasts. 45, 1434–1444. doi: 10.1007/s12237-021-01015-z

[B26] Lange M. Eisenhauer N. Sierra C. A. Bessler H. Engels C. Griffiths R. I.. (2015). Plant diversity increases soil microbial activity and soil carbon storage. Nat. Commun. 6, 6707. doi: 10.1038/ncomms7707 25848862

[B27] Lange M. Roth V.-N. Eisenhauer N. Roscher C. Dittmar T. Fischer-Bedtke C.. (2021). Plant diversity enhances production and downward transport of biodegradable dissolved organic matter. J. Ecol. 109, 1284–1297. doi: 10.1111/1365-2745.13556

[B28] Leimer S. Kreutziger Y. Rosenkranz S. Bessler H. Engels C. Hildebrandt A.. (2014). Plant diversity effects on the water balance of an experimental grassland. Ecohydrology 7, 1378–1391. doi: 10.1002/eco.1464

[B29] Li Y. Wang J. Shen C. Wang J. Singh B. K. Ge Y. (2022). Plant diversity improves resistance of plant biomass and soil microbial communities to drought. J. Ecol. 110, 1656–1672. doi: 10.1111/1365-2745.13900

[B30] Liang C. Cheng G. Wixon D. L. Balser T. C. (2011). An Absorbing Markov Chain approach to understanding the microbial role in soil carbon stabilization. Biogeochemistry 106, 303–309. doi: 10.1007/s10533-010-9525-3

[B31] Liang J.-F. Li Q.-W. Gao J.-Q. Feng J.-G. Zhang X.-Y. Hao Y.-J.. (2021). Biochar-compost addition benefits *Phragmites australis* growth and soil property in coastal wetlands. Sci. Total Environ. 769, 145166. doi: 10.1016/j.scitotenv.2021.145166 33486185

[B32] Liu J.-N. Wu F.-R. Roiloa S. R. Xue W. Lei N.-F. Yu F.-H. (2024). Emergent plant presence and richness alter competitive interactions between two floating plants. J. Plant Ecol. 17, rtae013. doi: 10.1093/jpe/rtae013

[B33] Loreau M. Hector A. (2001). Partitioning selection and complementarity in biodiversity experiments. Nature 412, 72–76. doi: 10.1038/35083573 11452308

[B34] Ma T. Deng X. Chen L. Xiang W. (2020). The soil properties and their effects on plant diversity in different degrees of rocky desertification. Sci. Total Environ. 736, 139667. doi: 10.1016/j.scitotenv.2020.139667 32485388

[B35] Ma M. Zhu Y. Wei Y. Zhao N. (2021). Soil nutrient and vegetation diversity patterns of alpine wetlands on the Qinghai-Tibetan Plateau. Sustainability 13, 6221. doi: 10.3390/su13116221

[B36] Martinez Richart A. I. Jimenez M. N. Fernandez Ondono E. Navarro F. B. (2019). Plant diversity in traditional irrigation channels and its relations with soil properties. Ecosistemas 28, 81–91. doi: 10.7818/ECOS.1733

[B37] Mellado-Vazquez P. G. Lange M. Bachmann D. Gockele A. Karlowsky S. Milcu A.. (2016). Plant diversity generates enhanced soil microbial access to recently photosynthesized carbon in the rhizosphere. Soil Biol. Biochem. 94, 122–132. doi: 10.1016/j.soilbio.2015.11.012

[B38] Mylliemngap W. Barik S. K. (2019). Plant diversity, net primary productivity and soil nutrient contents of a humid subtropical grassland remained low even after 50 years of post-disturbance recovery from coal mining. Environ. Monit. Assess. 191, 697. doi: 10.1007/s10661-019-7688-5 31989329

[B39] Poulter B. Fluet-Chouinard E. Hugelius G. Koven C. Fatoyinbo L. Page S. E.. (2021). A review of global wetland carbon stocks and management challenges. Am. Geophys. Union (AGU) pp, 1–20. doi: 10.1002/9781119639305.ch1

[B40] Prommer J. Walker T. W. N. Wanek W. Braun J. Zezula D. Hu Y.. (2020). Increased microbial growth, biomass, and turnover drive soil organic carbon accumulation at higher plant diversity. Glob. Change Biol. 26, 669–681. doi: 10.1111/gcb.14777 PMC7027739 31344298

[B41] Ratcliffe S. Wirth C. Jucker T. van der Plas F. Scherer-Lorenzen M. Verheyen K.. (2017). Biodiversity and ecosystem functioning relations in European forests depend on environmental context. Ecol. Lett. 20, 1414–1426. doi: 10.1111/ele.12849 28925074

[B42] Satdichanh M. Dossa G. G. O. Yan K. Tomlinson K. W. Barton K. E. Crow S. E.. (2023). Drivers of soil organic carbon stock during tropical forest succession. J. Ecol. 111, 1722–1734. doi: 10.1111/1365-2745.14141

[B43] Schultz R. Andrews S. O’Reilly L. Bouchard V. Frey S. (2011). Plant community composition more predictive than diversity of carbon cycling in freshwater wetlands. Wetlands 31, 965–977. doi: 10.1007/s13157-011-0211-6

[B44] Shannon C. E. Weaver W. (1949). The mathematical theory of communication, The mathematical theory of communication (Champaign, IL, US: University of Illinois Press).

[B45] Shen C. Wang J. Jing Z. Qiao N.-H. Xiong C. Ge Y. (2022). Plant diversity enhances soil fungal network stability indirectly through the increase of soil carbon and fungal keystone taxa richness. Sci. Total Environ. 818, 151737. doi: 10.1016/j.scitotenv.2021.151737 34808153

[B46] Simpson E. H. (1949). Measurement of diversity. Nature 163, 688. doi: 10.1038/163688a0

[B47] Spohn M. Bagchi S. Biederman L. A. Borer E. T. Brathen K. A. Bugalho M. N.. (2023). The positive effect of plant diversity on soil carbon depends on climate. Nat. Commun. 14, 6624–6624. doi: 10.1038/s41467-023-42340-0 37857640 PMC10587103

[B48] Steinbeiss S. Bessler H. Engels C. Temperton V. M. Buchmann N. Roscher C.. (2008). Plant diversity positively affects short-term soil carbon storage in experimental grasslands. Glob. Change Biol. 14, 2937–2949. doi: 10.1111/j.1365-2486.2008.01697.x

[B49] Thakur M. P. Milcu A. Manning P. Niklaus P. A. Roscher C. Power S.. (2015). Plant diversity drives soil microbial biomass carbon in grasslands irrespective of global environmental change factors. Glob. Change Biol. 21, 4076–4085. doi: 10.1111/gcb.13011 26118993

[B50] Tian F. Zhang Z. Chang X. Sun L. Wei X. Wu G. (2016). Effects of biotic and abiotic factors on soil organic carbon in semi-arid grassland. J. Soil Sci. Plant Nutr. 16, 1087–1096. doi: 10.4067/S0718-95162016005000080

[B51] Token S. Jiang L. Zhang L. Ly G. (2022). Effects of plant diversity on primary productivity and community stability along soil water and salinity gradients. Glob. Ecol. Conserv. 36, e02095. doi: 10.1016/j.gecco.2022.e02095

[B52] Vance E. D. Brookes P. C. Jenkinson D. S. (1987). An extraction method for measuring soil microbial biomass C. Soil Biol. Biochem. 19, 703–707. doi: 10.1016/0038-0717(87)90052-6

[B53] Wrang X. Dong S. Yang B. Li Y. Su X. (2014). The effects of grassland degradation on plant diversity, primary productivity, and soil fertility in the alpine region of Asia’s headwaters. Environ. Monit. Assess. 186, 6903–6917. doi: 10.1007/s10661-014-3898-z 25023744

[B54] Wang C. Hou Y. Hu Y. Zheng R. Li X. (2023). Plant diversity increases above- and below-ground biomass by regulating multidimensional functional trait characteristics. Ann. Bot. 131, 1001–1010. doi: 10.1093/aob/mcad058 37119271 PMC10332393

[B55] Wang C. Ma L. Zuo X. Ye X. Wang R. Huang Z.. (2022). Plant diversity has stronger linkage with soil fungal diversity than with bacterial diversity across grasslands of northern China. Glob. Ecol. Biogeogr. 31, 886–900. doi: 10.1111/geb.13462

[B56] Wang X. Wang J. Hu B. Zheng W.-L. Li M. Shen Z.-X.. (2022). Richness, not evenness, of invasive plant species promotes invasion success into native plant communities via selection effects. Oikos 2022, e08966. doi: 10.1111/oik.08966

[B57] Wang S. Zhao S. Yang B. Zhang K. Fan Y. Zhang L.. (2022). The carbon and nitrogen stoichiometry in litter-soil-microbe continuum rather than plant diversity primarily shapes the changes in bacterial communities along a tropical forest restoration chronosequence. Catena 213, 106202. doi: 10.1016/j.catena.2022.106202

[B58] Wu X. Yao Z. Brueggemann N. Shen Z. Y. Wolf B. Dannenmann M.. (2010). Effects of soil moisture and temperature on CO2 and CH4 soil atmosphere exchange of various land use/cover types in a semi-arid grassland in Inner Mongolia, China. Soil Biol. Biochem. 42, 773–787. doi: 10.1016/j.soilbio.2010.01.013

[B59] Xi N. Chen D. Liu W. Bloor J. M. G. (2023). Positive plant diversity effects on soil microbial drought resistance are linked to variation in labile carbon and microbial community structure. Funct. Ecol. 37, 2347–2357. doi: 10.1111/1365-2435.14396

[B60] Xu Y. Ge X. Gao G. Yang Y. Hu Y. Li Z.. (2023). Divergent contribution of microbial- and plant-derived carbon to soil organic carbon in Moso bamboo forests left unmanaged. Catena 233, 107481. doi: 10.1016/j.catena.2023.107481

[B61] Xue W. Yao S.-M. Huang L. Roiloa S. R. Ji B.-M. Yu F.-H. (2022). Current plant diversity but not its soil legacy influences exotic plant invasion. J. Plant Ecol. 15, 639–649. doi: 10.1093/jpe/rtab065

[B62] Yan M. Cui F. Liu Y. Zhang Z. Zhang J. Ren H.. (2020). Vegetation type and plant diversity affected soil carbon accumulation in a postmining area in Shanxi Province, China. Land Degrad. Dev. 31, 181–189. doi: 10.1002/ldr.3438

[B63] Yang L. Jia W. Shi Y. Zhang Z. Xiong H. Zhu G. (2020). Spatiotemporal differentiation of soil organic carbon of grassland and its relationship with soil physicochemical properties on the northern slope of Qilian Mountains, China. Sustainability 12, 9396. doi: 10.3390/su12229396

[B64] Yao B. Wang X. Li Y. Lian J. Li Y. Luo Y.. (2023). Soil extracellular enzyme activity reflects the change of nitrogen to phosphorus limitation of microorganisms during vegetation restoration in semi-arid sandy land of northern China. Front. Environ. Sci. 11. doi: 10.3389/fenvs.2023.1298027

[B65] Zhang Y. Duan B. Xian J. Korpelainen H. Li C. (2011). Links between plant diversity, carbon stocks and environmental factors along a successional gradient in a subalpine coniferous forest in Southwest China. For. Ecol. Manage. 262, 361–369. doi: 10.1016/j.foreco.2011.03.042

[B66] Zhang Z. Jiang W. Peng K. Wu Z. Ling Z. Li Z. (2023). Assessment of the impact of wetland changes on carbon storage in coastal urban agglomerations from 1990 to 2035 in support of SDG15.1. Sci. Total Environ. 877, 162824. doi: 10.1016/j.scitotenv.2023.162824 36948315

[B67] Zhao Q. Bai J. Wang X. Zhang W. Huang Y. Wang L.. (2020). Soil organic carbon content and stock in wetlands with different hydrologic conditions in the Yellow River Delta, China. Ecohydrol. Hydrobiol. 20, 537–547. doi: 10.1016/j.ecohyd.2019.10.008

[B68] Zhao R. Zhang X. Zhang L. Wang Y. (2021). Plant diversity and soil properties at different wetland restoration stages along a major river in the arid northwest of China. Wetlands 41, 13. doi: 10.1007/s13157-021-01412-x

[B69] Zheng H. Liu D. Yuan J. Li Y. Li J. Miao Y.. (2024). Wetland restoration after agricultural abandonment enhances soil organic carbon efficiently by stimulating plant- rather than microbial-derived carbon accumulation in Northeast China. Catena 241, 108077. doi: 10.1016/j.catena.2024.108077

[B70] Zhou P. Zhang L. Qi S. (2022). Plant diversity and aboveground biomass interact with abiotic factors to drive soil organic carbon in beijing mountainous areas. Sustainability 14, 10655. doi: 10.3390/su141710655

[B71] Zou J.-Y. Luo Y.-H. Seidl R. Thom D. Liu J. Geres L.. (2024). No generality in biodiversity-productivity relationships along elevation in temperate and subtropical forest landscapes. For. Ecosyst. 11, 100187. doi: 10.1016/j.fecs.2024.100187

