# Supplementary figures and images for: Increasing plant diversity enhances soil organic carbon storage in typical wetlands of northern China
Source: Front Plant Sci. 2024 Dec 2;15:1467621. doi: 10.3389/fpls.2024.1467621 (PMC11646718; doi:10.3389/fpls.2024.1467621)

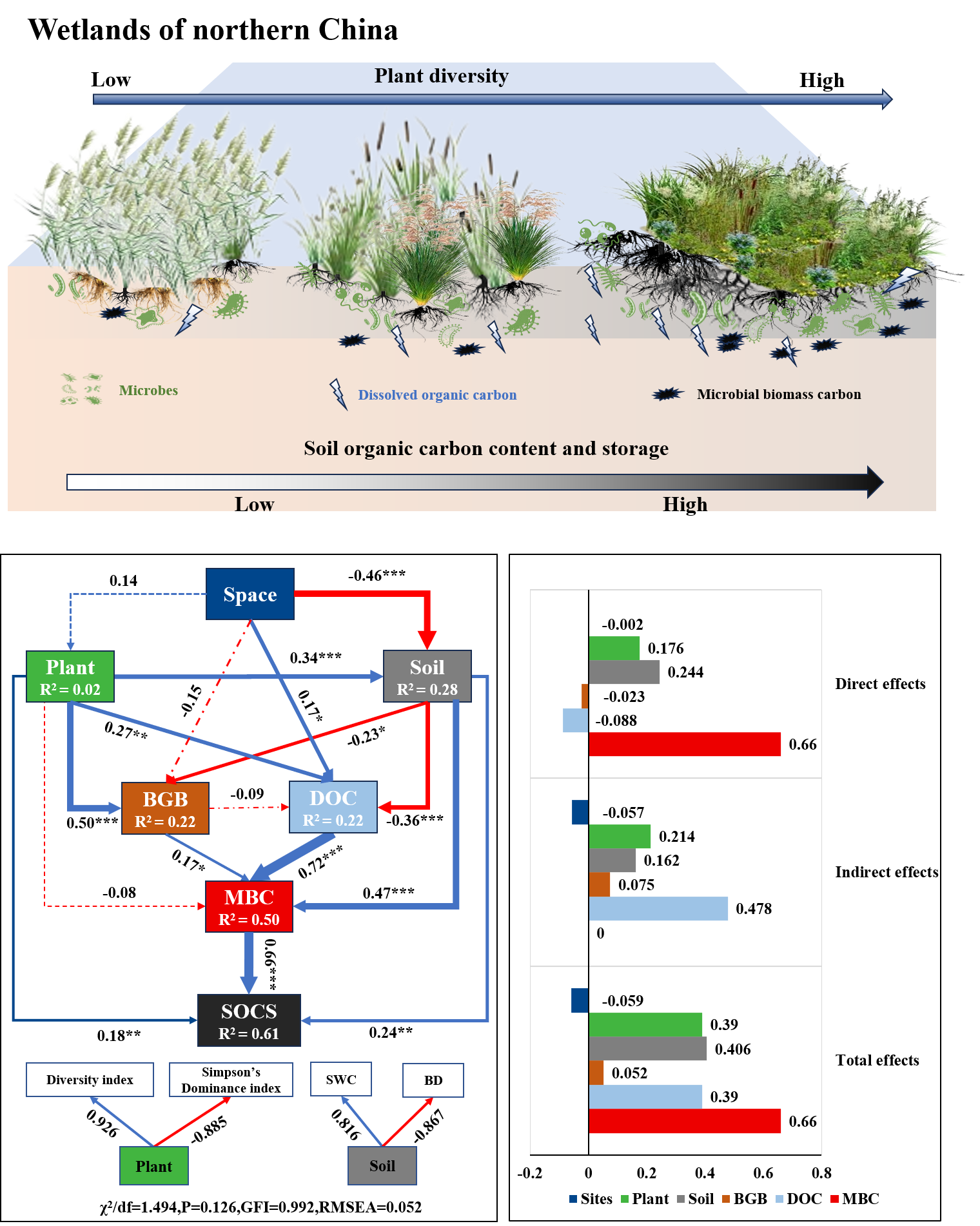

Supplement: Supplementary file 1 [file DataSheet1.docx]
